# Recent Trends in Economic Burden of Acute Myocardial Infarction in South Korea

**DOI:** 10.1371/journal.pone.0117446

**Published:** 2015-02-06

**Authors:** Hyeyoung Seo, Seok-Jun Yoon, Jihyun Yoon, Dongwoo Kim, Younghoon Gong, A. Rim Kim, In-Hwan Oh, Eun-Jung Kim, Yo-Han Lee

**Affiliations:** 1 Department of Public Health, Graduate School, Korea University, Seoul, Republic of Korea; 2 Department of Preventive Medicine, College of Medicine, Korea University, Seoul, Republic of Korea; 3 Department of Preventive Medicine, School of Medicine, Kyung Hee University, Seoul, Republic of Korea; 4 Economic Research Institute, Korea University, Seoul, Republic of Korea; Azienda Ospedaliero-Universitaria Careggi, ITALY

## Abstract

In 2010, ischemic heart disease was the leading cause of disability-adjusted life-years (DALYs) worldwide. More specially, the prevalence of acute myocardial infarctions (AMI) is increasing in the aged population as mortality decreases; South Korea is no exception. This study aims to examine the economic burden of AMI in the Korean population between 2007 and 2012. AMI-related costs were assessed from a societal perspective. A prevalence-based cost-of-illness framework was used for this analysis. The subjects included all South Koreans with AMI-related ICD-10 codes (I21, I22, I23, I25.0, and I25.1). Data on direct (medical and non-medical) costs and indirect (productivity loss due to AMI-associated morbidity and mortality) costs were collected from the Korean National Health Insurance Service’s claims data. The human capital approach was used to calculate indirect costs. The total estimated cost of AMI in 2012 was $1,177,649,323 USD. The majority (52%) of this amount was made up of medical costs, followed by productivity losses due to mortality and morbidity (42% of annual cost). Although the total cost declined by approximately 18% compared to 2007 ($1,427,643,854 USD), the cost of AMI in the over 60 age group amounted to 47% of the total cost of AMI in 2012. AMI led to a high economic burden in 2012. This study, which identified not only the size, but also the trends of AMI-related costs, will provide information to evaluate effects of governmental health projects and the effective allocation of public research funds.

## Introduction

Acute myocardial infarction (AMI) is a disease associated with high mortality worldwide [1]. However, since survival rates of patients with ischemic heart diseases (IHD) such as AMI are increasing, IHD is the leading cause of disability-adjusted life-years (DALYs) [1, 2]. According to the Korean Statistical Information Service (KOSIS), the number of deaths due to AMI (I21) was 19.6 out of every 100,000 Koreans in 2012 [3]. Also, while the AMI-associated mortality has gradually decreased, the prevalence of AMI is on the rise due to improvement in the early detection and treatment of AMI [4]. AMI has a heavy socioeconomic burden. Healthcare costs related to disease morbidity are most closely associated with the prevention and management of disease, directly. In addition, AMI not only prevents many patients from working for their living, but also renders them dependent on caregivers throughout the duration of disease morbidity. Therefore, when we measure the burden of AMI, we need to take into account loss of productivity and caregiver costs as well as direct healthcare costs related to treatment.

This cost-of-illness (COI) study provides valuable data, which is comparable to the relative socioeconomic burdens of other disease. It can also be used as objective evidence when policy makers establish policy priorities and allocate resources related to the healthcare system. The objectives of this study were to quantify the economic burden of AMI caused by an increased in the use of health resources in combination with lost productivity between 2007 and 2012, and to provide a detailed breakdown of the costs attributed to AMI by various sex- and age-related groups.

## Materials and Methods

### Analysis framework and data sources

This study evaluated the costs related to AMI in a population-based cost analysis. We used a prevalence-based approach and a macro-costing method. AMI is defined here by the World Health Organization International Classification of Diseases (10^th^ revision) codes I21 (acute MI), I22 (subsequent MI), I23 (certain current complication following acute MI), I25.0 (atherosclerotic cardiovascular disease, so described), and I25.1 (atherosclerotic heart disease). Though I25.0 and I25.1 are associated with chronic conditions, we have included those codes in our study in an effort to reflect typical Korean features related to entering AMI codes. In Korea, several medical institutions frequently make claims for health insurance premiums using codes I25.0 and I25.1 when they conduct screening tests (a creatine kinase muscle-brain (CK-MB) and troponin) and administer treatments (coronary artery bypass graft (CABG), percutaneous coronary intervention (PCI)) to AMI patients. Additionally, a previous study showed that the positive rate of MI associated with both codes I25.0 and I25.1 was high [5]. Therefore, we included codes I25.0 and I25.1 not only to prevent underestimating prevalence of AMI, but also to increase the comparability of our data with previous studies.

We defined AMI patients as those who had records of outpatient treatment or hospitalization due to AMI more than once in a year. When analyzing the claims data by main disease and first sub disease of patients diagnosed with AMI, we selected claims from the available data that were made in reference to AMI codes, I21, I22, I23, I25.0, and I25.1. Next, we dropped any claims with cancer diagnoses (ICD-10 code: C00-C97) from the set. The intention behind dropping this data was to prevent an overestimation of expenses related to AMI by excluding comorbidity diseases, which often cost a great deal to treat [6].

We estimated direct costs (medical costs and non-medical costs) and indirect costs (productivity losses due to morbidity and mortality) based on a societal perspective.

AMI medical costs were divided into three categories: health insurance costs, out-of-pocket medical expenses and drugs. We used health insurance claims data to calculate health insurance costs related AMI throughout 2007 and 2012. Because there is only one insurance system in Korea, the National Health Insurance Service (NHIS) data represents medical information of the entire South Korean population. To account for private expenditures, we used data from health insurance patients’ medical expenditure surveys from 2007 and 2012 [7]. Drug expenditure was estimated by using NHIS claims data and health insurance statistical yearbook [8]. Non-medical costs included informal care costs, which were comprised of transportation costs and caregiver costs. We used the Korean National Health and Nutrition Examination Survey (KNHNES) data to estimate transportation costs [9] and caregiver costs were calculated using city’s daily workers’ wage data announced by the Construction Association of Korea and the Korea Federation of Small and Medium Business [10]. In Korea, when private insurance holders make a claim for caregiver costs due to injury or other such reasons, the insurer pays caregiver expenses according to daily workers’ wage in cities. Therefore, this study also used the wages to estimate caregiver costs.

We used a human-capital approach to estimate indirect (productivity loss) costs [11]. Productivity costs included the foregone earnings of patients with AMI-attributable mortality and morbidity. We acquired average daily wage and employment rate data from the Employment and Labor Ministry [12]. We also considered the number of deaths by specific causes to estimate costs of lost productivity due to AMI mortality. Cause-of-death data were established from micro-data service system of KOSIS [13].

We use an annual time frame to assess change in economic burden between 2007 and 2012. The results of epidemiologic analysis (prevalence and mortality) were presented as the trends from 2007 to 2012 and the total cost of AMI was presented by comparing with the results in 2007 and 2012. Though this study used the same basic framework as has previously been used to measure the costs of chronic diseases [14, 15], employment rate was also considered in the estimate of direct costs. Therefore, when we compare the economic burden of this disease over time, the probability of overestimation in previous estimates should be considered. This study did not require approval of an Institutional Review Board. All costs in the study are expressed as they were recorded in 2007 and 2012 and have been converted to USD.

### Procedures and statistical analysis

Direct costs include medical costs and non-medical costs. Medical utilization has been divided into three categories: outpatient care, hospital inpatient care and drugs. Non-medical costs include transportation costs and caregiver costs.

Medical costs consisted of reimbursement from NHIS and out-of-pocket expenses. First, we calculated both outpatient and inpatient reimbursement using NHIS claims data. Next, using the proportion of out-of-pocket costs for patients with heart disease (I05–I15, I20–I28, I30–52) categorized by treatment type in the NHIS patient medical costs survey, we estimated out-of-pocket expenses. Once reimbursement and out-of-pocket expenses for outpatient and inpatient care were estimated, the total medical cost was added up.

In case of drug expenditures, inpatients costs already included drug costs incurred during hospitalization. Therefore, we only needed to consider outpatients’ drug expenses, which were calculated using the proportion of outpatient, inpatient and outpatients’ drug costs provided by an annual NHIS statistical report [8].

$$Medical\;cost=\underset{ij}{\sum }\sum^{\text{​}}\{({\text{InNHI}}_{ij}+{\text{OP}}_{ij})+({\text{OutNHI}}_{ij}+{\text{OP}}_{ij})\}+{\text{OutD}}_{ij}$$

*InNHI = NHI payment for hospitalization expenditures, OutNHI = NHI payment for outpatient expenditure, OP = Out-of-pocket payment, OutD = Outpatient payment for Drug, i = 0, 1, …, n (age), and j = 1 or 2 (sex)*

We defined non-medical costs as transportation costs and caregiver costs. To estimate the transportation costs, we multiplied the number of visits to a medical institution for AMI treatment by travel expense per visit. We used a single fare per visit to outpatient and inpatient institutions from KNHNES in 2005, and final transportation expenditures were estimated in consideration of inflation rates each year. Caregiver costs incurred through patients’ length-of-stay in hospitals and the hours of visits for outpatient care-related AMI were also estimated. The caregiver costs during hospitalization were calculated by multiplying patients’ length-of-stay in hospital by the average city daily workers’ wage. When we estimated the caregiver costs related to outpatient visits, we assumed that patients under the age of 20 and over the age of 70 would require a guardian and approximately 8 hours would be needed to accompany the patient to outpatient services.

$$\mathrm{Non}-\mathrm{medical}\;\mathrm{cost}=\underset{ij}{\sum^{\text{​}}}{\sum^{\text{​}}}^{\text{​}}\left\{\left({\text{InV}}_{ij}\times {\text{InT}}_{ij})+({\text{OutV}}_{ij}\times {\text{OutT}}_{ij}\right)\right\}+\left\{({\text{InD}}_{ij}\times \text{CG})+\left({\text{OutV}}_{ij}\times \text{CG}\times \frac{1}{3}\right)\right\}-\underset{j}{\sum^{\text{​}}}\overset{69}{\underset{i=20}{\sum^{\text{​}}}}\left({\text{OutV}}_{ij}\times \text{CG}\times \frac{1}{3}\right)$$

*InV = number of hospitalization visits, OutV = number of outpatient visits, InT = round-trip transportation costs per hospitalization visit, OutT = round-trip transportation costs per outpatient visit, InD = length of hospitalization, CG = caregiver costs per day, i = 0, 1, …, n (age), and j = 1 or 2 (sex)*

Indirect costs included lost productivity due to AMI-related mortality and morbidity. We estimated productivity costs attributable to mortality as earnings lost due to premature death. For that, we assumed that the ages of the economically productive population were between 15 and 69 years. To estimate productivity loss after premature death due to AMI, we multiplied the number of deaths (categorized by sex and age) by annual average income. We also took the employment rate at the time into account to estimate the final value.

$$\text{Productivity losses due to mortality}=\underset{i}{\sum^{\text{​}}}\underset{j}{\sum^{\text{​}}}\overset{n}{\underset{k}{\sum^{\text{​}}}}({\text{N}}_{ij}\times \frac{{\text{Y}}_{ij\left(\text{t}+\text{k}\right)}\times {\text{P}}_{ij\left(\text{t}+\text{k}\right)}}{{\left(1+\text{r}\right)}^{k}})$$

*N = number of death, Y_t+k_ = average annual income at (t+k), P_t+k_ = employment rate, t = age at death, k = 1, 2, …, n (n is the difference between life expectancy and age at the time of death), r = discount rate, i = 0, 1, …, n (age), and j = 1 or 2 (sex)*

Morbidity costs due to AMI were calculated and divided into two categories, outpatient and inpatient. We multiplied patients’ length-of-stay in hospital by the daily average wage (in terms of sex and age) to estimate productivity loss due to AMI inpatient care. Though productivity loss related to outpatient care was calculated in the same way, for the purposes of this study, we assumed that 8 hours of productivity was lost due to each outpatient care visit.

$$\text{Productivity losses due to morbidity}=\sum^{\text{​}}\underset{ij}{\sum }\{({\text{InD}}_{ij}\times {\text{ IC}}_{ij}\times {\text{ E}}_{ij})+\left({\text{OutV}}_{ij}\times \frac{1}{3}\times {\text{ IC}}_{ij}\times {\text{E}}_{ij}\right)\}$$

*InD = length of hospitalization, IC = average daily income, E = employment rate, OutV = number of outpatient visits, i = 0, 1, …, n (age), and j = 1 or 2 (sex)*

## Results

The prevalence of AMI continued to rise during the study period. In terms of sex, the number of male patients with AMI (149,648) is more than 1.73 times the number of females (86,691) in 2012. Even the increase in patients with AMI between 2007 and 2012 includes more male (1.34 times) than female (1.13 times) patients. Mortality due to AMI (per 100,000 of the population) decreased further each year from 2007 to 2011. However, the number of deaths due to AMI rose in 2012 to a number similar to that of 2008. In terms of the number of deaths due to AMI by age groups, mortality continued to decline 0.80 times in the under 60 age group between 2007 and 2012 (Table 1).

**Table 1:** Epidemiology of acute myocardial infarction in South Korea from 2007 to 2012.

** **	**2007**	**2008**	**2009**	**2010**	**2011**	**2012**	**Growth rate in 2012 over 2007**
***Prevalence***							
**Number of patients**	188,493	191,424	201,296	222,662	227,316	236,339	1.25
**Prevalence per 1000 population**	3.83	3.86	4.04	4.41	4.48	4.64	
**Gender**							
Male	111,899	115,416	122,594	136,625	141,764	149,648	1.34
Female	76,594	76,008	78,702	86,037	85,552	86,691	1.13
**Age**							
Under 60	70,376	70,619	76,325	85,822	88,457	93,566	1.33
Over 60	118,117	120,805	124,971	136,840	138,859	142,773	1.21
***Mortality***							
**Number of deaths**	11,151	10,036	9,903	9,990	9,878	10,549	0.95
**Mortality per 100,000 population**	22.63	20.26	19.90	19.78	19.47	20.71	
**Gender**							
Male	6,096	5,609	5,537	5,487	5,358	5,745	0.94
Female	5,055	4,427	4,366	4,503	4,520	4,804	0.95
**Age**							
Under 60	2,022	1,908	1,801	1,675	1,572	1,625	0.80
Over 60	9,129	8,128	8,102	8,315	8,306	8,924	0.98

We estimated the total economic cost of AMI in South Korea to be more than $1,177,649,323 USD in 2012 (Table 2). This accounts for an approximate 18% decline in AMI costs compared with those of 2007 ($1,427,643,854 USD). While the proportion of direct costs and indirect costs compared to the total costs was balanced in 2007, direct costs (58%) grew more than indirect costs in 2012, in proportion to total costs. The majority of AMI-related medical costs is attributed to inpatient care, which accounted for $442,138,977 USD, representing 73% of total medical costs in 2012. Non-medical costs in 2012 had grown approximately 23% since 2007 and the leading cause of this rise was increasing caregiver costs. The productivity losses attributable to AMI mortality and morbidity declined by $237,425,381 USD (32%) from 2007 to 2012, and this reduction was led by a decline in AMI-related mortality. According to the costs by sex, male AMI costs were more than 3 times higher than female’s cost in both 2007 and 2012 years. Additionally, the economic burden of AMI in the over 60 age group represented approximately 47% of the total costs in 2012 (Table 2).

**Table 2:** Costs of acute myocardial infarction in South Korea between 2007 and 2012 (Unit: USD million).

** **	**2007**			**2012**		
	**Male**	**Female**	***Total (%)***	**Male**	**Female**	***Total (%)***
***Direct costs***	*435.92*	*255.80*	*691.72 (48.45)*	*440.82*	*238.33*	*679.15 (57.67)*
**Medical costs**	404.39	229.12	633.51 (44.37)	402.69	204.59	607.28 (51.57)
Inpatient	281.85	158.15	440.00	293.68	148.45	442.14
Outpatient	122.54	70.97	193.51	109.00	56.14	165.14
**Non-medical costs**	31.53	26.68	58.21 (4.08)	38.13	33.74	71.87 (6.10)
Transportation	3.26	2.15	5.41	3.66	2.15	5.81
Caregiving	28.27	24.53	52.80	34.47	31.59	66.06
***Indirect costs (Productivity losses)***	*675.54*	*60.39*	*735.92 (51.55)*	*467.74*	*30.75*	*498.50 (42.33)*
**Mortality**	657.90	53.00	710.90 (49.80)	433.96	27.01	460.97 (39.14)
**Morbidity**	17.64	7.39	25.02 (1.75)	33.78	3.74	37.53 (3.19)
***Total costs***	*1,111.45*	*316.19*	*1,427.64 (100.00)*	*908.56*	*269.08*	*1,177.65 (100.00)*
**Over 60 year-old age group**	348.90	233.78	582.67 (40.81)	335.97	217.07	553.04 (46.96)

$1 USD = 929 won in 2007; $1USD = 1,126 won in 2012.

## Discussion

We estimated the total cost of AMI in South Korea at to be $1.178 billion USD in 2012, of which, $607 million USD was incurred by direct medical utilization. The other 48% of the economic burden of AMI, however, was incurred in non-health-care systems, with almost $461 million USD in lost productivity attributable to early death. The total costs due to AMI accounted for 1.28% of National Health Expenditure (NHE) in 2012 and this cost showed a 0.7% decrease from the figure in 2007 [16]. The proportion of AMI costs from National Health Insurance records also decreased 5.12% in 2007 to 3.39% in 2012 [8].

Total AMI costs had decreased in 2012 compared with the costs in 2007. We can predict that the decline of the economic burden due to AMI happened since the decreasing rate of AMI mortality exceeded the growth rate of AMI prevalence. In addition, the decline in AMI mortality may be caused mostly by improvements in acute cardiac treatment [17]. However, inpatient care costs have climbed for the last 6 years, which may be attributed to not only a growth disease incidence, but also a higher recurrence rate as a consequence of longer survival after the first coronary onset [17, 18]. In a rapidly aging society, the number of patients with higher severity AMI are likely to increase [19–21], which can lead to an increase in medical expenditures related to hospitalization. Therefore, not only primary and secondary prevention will play the main roles in preventing increasing in severity of AMI, by expending public support for AMI-related inpatient care and caregiver service, the burden of patients with AMI will hopefully decrease [17, 18].

According to the economic burden by sex, male AMI costs were higher than female’s costs. Especially, direct costs were 1.85 times and indirect costs (productivity losses) were 15.21 times higher than females in 2012. The higher costs in male were caused by both higher prevalence and mortality of AMI than females. The AMI-related risk factors in males were reported to be smoking and stress [17, 22, 23]. Therefore, to decrease the economic burden of AMI, an active intervention strategy should be established to manage these risk factors in male AMI patients.

Recently, cost-of-illness studies have continued to progress in order to estimate the impact of diseases with similar frameworks in Korea. Stroke burden evaluations in 2010 estimated direct costs to be $3.528 billion USD, which was much higher than the direct cost of AMI in our 2012 evaluation [24]. Additionally, the economic costs of coronary heart diseases (CHD), including AMI, were estimated at $2.518 billion USD, and 38% of this cost ($954 million USD) was attributed to myocardial infarction [25]. Due to these results, we can not only predict the relative scale of current CHD costs in 2012, but also demonstrate the need for prevention and management strategies for cardiovascular diseases [26]. By comparing the estimated economic burden of multiple diseases using this standardized framework, policy makers can make informed decisions about the allocation of resources to service provision, prevention strategies, and research funding [27].

We found that in other developed countries, the distribution of AMI economic burden was similar to that of our study [19, 28–30]. Coronary heart disease exacted a high toll in terms of direct, inpatient costs, as well as indirect, productivity losses due to mortality. As these components depend on initial treatment, readmission rate, and population’s age structure, clinical studies strongly suggest that the health care systems need improvement to provide efficient, safe and cost-effective treatment by developing management strategy based on risk-stratification of the patient and optimal medical therapy guideline.

Even though disease-related costs were estimated, we cannot discuss whether a country is spending too much or too little on a disease. Rather, the main aim of a COI study is to help inform decisions concerning allocation of research funding, by providing a measure of the economic burden of particular health problems [31]. In addition, by implementing COI studies periodically, we not only stay informed about the trends of economic burden, but also use datasets as a tool to evaluate the effects of changing policies. In Korea, many projects have been established at the national-level, such as implementation of educational and promotional programs, management of risk factors (hypertension, diabetes), improvement of disease (stroke and MI) management levels, and the establishment of infrastructure for management of cardiovascular diseases through cardiovascular disease prevention strategies. The aim of these projects is to improve national health status through preventive efforts [32]. Therefore, this COI study will be a useful tool to evaluate effects of national-level projects [26].

We used NHIS claims data as a main data source to estimate the cost of AMI. Since NHIS claims data include most of information necessary to calculate direct and indirect costs, such as main and sub disease codes, NHI costs, distinguishable variables for inpatient or outpatient care, and other variables, this data source provided key information. In addition, since statistics of mortality from the Korea National Statistical Office provide information about cause of death, we are confident that number of deaths-by-cause reported in this study is accurate. KNHNES data, which was used to calculate transportation expenses, has been established by the Korea Center for Disease Control and Prevention and is comprised of survey data based on a representative, extracted sample. To calculate caregiver costs, we used the public notice amount as the estimated daily worker’s wage based on legal criteria for payment of caregivers. The variables related to indirect costs calculations, such as daily average wage, annual work days and employment rate, were derived from statistics on employment and labor by the Ministry of Employment and Labor. Therefore, the data used in this study was established based on representative data sources.

It is important to note that our study had several limitations. First, our estimates are likely to be low, or conservative. We could not include some costs related to the healthcare expenditures, such as public health promotion activities and health education due to data source limitation. However, other studies have shown that these costs represent a small proportion of total costs [33, 34]. Furthermore, we cannot consider rates of employment loss due to AMI-morbidity and employment return rate after morbidity because of data source limitations. Finally, since the employment status of patients can change due to AMI severity, we need to consider that in future studies.

Our study is significant because it analyzed trends over the last six years of economic burden and the epidemiological index of AMI using recent data. These analyses demonstrate the importance of improving the quality of disease management, not only to promote awareness of the current epidemiologic status of this disease, but also so that decision makers can implement evidence-based policies. In this respect, our study provides reliable COI data and indirectly reflects the performance of national projects related to prevention of cardiovascular disease. We believe that our study will offer meaningful information to the decision makers of the Korean government.
